# Clinician-Based Functional Scoring and Genomic Insights for Prognostic Stratification in Wolf–Hirschhorn Syndrome

**DOI:** 10.3390/genes16070820

**Published:** 2025-07-12

**Authors:** Julián Nevado, Raquel Blanco-Lago, Cristina Bel-Fenellós, Adolfo Hernández, María A. Mori-Álvarez, Chantal Biencinto-López, Ignacio Málaga, Harry Pachajoa, Elena Mansilla, Fe A. García-Santiago, Pilar Barrúz, Jair A. Tenorio-Castaño, Yolanda Muñoz-GªPorrero, Isabel Vallcorba, Pablo Lapunzina

**Affiliations:** 1 Instituto de Genética Médica y Molecular (INGEMM)-IdiPAZ, Hospital Universitario La Paz, 28046 Madrid, Spain; mangeles.mori@salud.madrid.org (M.A.M.-Á.); elena.mansilla@salud.madrid.org (E.M.); feamalia.garcia@salud.madrid.org (F.A.G.-S.); pilarbarruz@gmail.com (P.B.); jaira.tenorio@salud.madrid.org (J.A.T.-C.); ymunozg@salud.madrid.org (Y.M.-G.); isabel.vallcorba@salud.madrid.org (I.V.); plapunzina@gmail.com (P.L.); 2 CIBERER, Centro de Investigación Biomédica en Red de Enfermedades Raras, ISCIII, 28046 Madrid, Spain; 3 ITHACA-European Reference Network-Hospital La Paz, 28046 Madrid, Spain; 4 Facultad HM de Ciencias de la Salud, Universidad Camilo José Cela e Instituto de Investigación Sanitaria HM Hospitales, 28015 Madrid, Spain; 5 Servicio de Neuropediatría, Hospital Universitario Central de Asturias (HUCA), 33011 Oviedo, Spain; rablabul81@gmail.com (R.B.-L.); neuropediatria.huca@gmail.com (I.M.); 6 Departamento de Investigación y Psicología en Educación, Facultad de Educación, Universidad Complutense de Madrid, 28040 Madrid, Spain; mbel@ucm.es (C.B.-F.); alameda@edu.ucm.es (C.B.-L.); 7 Departamento de Economía Financiera y Actuarial y Estadística, Facultad de Comercio y Turismo, Universidad Complutense de Madrid, 28040 Madrid, Spain; adolfher@ucm.es; 8 Centro de Investigaciones en Anomalías Congénitas y Enfermedades Raras, Universidad ICESI, Cali 760031, Colombia; hmpachajoa@icesi.edu.co

**Keywords:** epilepsy, 4p16.3, developmental delay, Wolf–Hirschhorn syndrome, functional assessment, COA

## Abstract

**Background/Objectives**: Wolf–Hirschhorn syndrome (WHS; OMIM #194190) is a rare neurodevelopmental disorder, caused by deletions in the distal short arm of chromosome 4. It is characterized by developmental delay, epilepsy, intellectual disability, and distinctive facial dysmorphism. Clinical presentation varies widely, complicating prognosis and individualized care. **Methods**: We assembled a cohort of 140 individuals with genetically confirmed WHS from Spain and Latin-America, and developed and validated a multidimensional, Clinician-Reported Outcome Assessment (ClinRO) based on the Global Functional Assessment of the Patient (GFAP), derived from standardized clinical questionnaires and weighted by HPO (Human Phenotype Ontology) term frequencies. The GFAP score quantitatively captures key functional domains in WHS, including neurodevelopment, epilepsy, comorbidities, and age-corrected developmental milestones (selected based on clinical experience and disease burden). **Results**: Higher GFAP scores are associated with worse clinical outcomes. GFAP showed strong correlations with deletion size, presence of additional genomic rearrangements, sex, and epilepsy severity. Ward’s clustering and discriminant analyses confirmed GFAP’s discriminative power, classifying over 90% of patients into clinically meaningful groups with different prognoses. **Conclusions**: Our findings support GFAP as a robust, WHS-specific ClinRO that may aid in stratification, prognosis, and clinical management. This tool may also serve future interventional studies as a standardized outcome measure. Beyond its clinical utility, GFAP also revealed substantial social implications. This underscores the broader socioeconomic burden of WHS and the potential value of GFAP in identifying high-support families that may benefit from targeted resources and services.

## 1. Introduction

Wolf–Hirschhorn syndrome (WHS; OMIM #194190) is a rare genetic disorder resulting from partial deletions on the short arm of chromosome 4, particularly within the 4p16.3 region [1,2,3]. The size of the deletion can vary between 700 Kb to 35 Mb, which includes the *WSHC1* (*NSD2*) gene within the putative main responsible region for the syndrome [4,5,6,7,8]. The clinical phenotype includes growth and developmental delay, intellectual disability, seizures, microcephaly, and distinct craniofacial features commonly described as a “Greek warrior helmet” profile. The syndrome has an estimated prevalence of 1 in 20,000 to 50,000 live births and is more frequently diagnosed in females than in males. Our most recent work with the Spanish individuals of this cohort established a frequency of 1/172,904 newborns [9], suggesting that this syndrome may be underdiagnosed, as it occurs in other near countries [10]. While the causative genetic alterations are well established, the wide spectrum of phenotypic variability remains poorly understood, posing challenges for clinical management and prognostic counseling.

Existing clinical guidelines for WHS emphasize the need for multidisciplinary follow-up; however, standardized tools for assessing disease severity and monitoring patient progress over time are lacking. Clinical Outcome Assessments (COAs), particularly those developed for rare diseases, have demonstrated value in providing structured evaluations of patient status and in guiding treatment decisions [11,12,13,14,15,16,17,18,19]. In WHS, no syndrome-specific COA currently exists. This gap limits our ability to objectively evaluate interventions and to offer families reliable information about likely disease trajectories. Indeed, there are neither enough data on adulthood, life expectancy, nor longitudinal work in these patients [20,21,22,23,24,25,26] that helps clinicians to adequately monitor and predict their evolution.

To address this need, we developed a disease-specific Clinician-Reported Outcome (ClinRO) tool for WHS: the Global Functional Assessment of the Patient (GFAP). This measure integrates multiple domains of patient function, including developmental milestones, epilepsy characteristics, and comorbidities, to provide a composite score reflecting overall clinical severity. The GFAP was constructed using data from one of the largest and most clinically diverse WHS cohorts (140 individuals) to date, incorporating individuals from both Spain and Latin America. Our previous work in this syndrome was taken as sufficient to achieve expertise and in-depth understanding of the medical aspects of the knowledge of the concept of interest (COI). Here, we present the development and validation of the GFAP score and its utility in identifying phenotypic subgroups within WHS. We demonstrate its association with key clinical and genetic features and evaluate its discriminatory capacity through cluster and discriminant analysis.

## 2. Materials and Methods

### 2.1. Cohort Description

**Figure 1:**
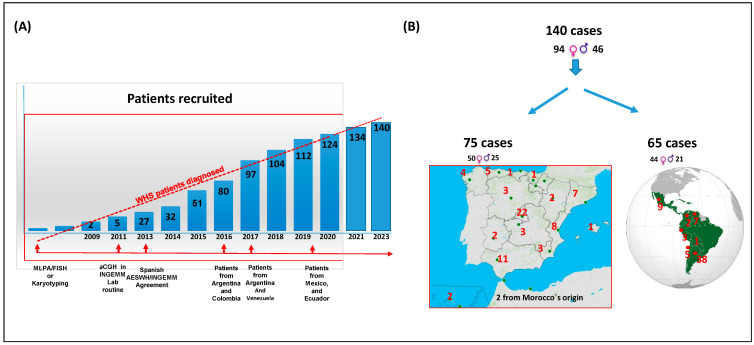
Recruitment of the cohort, and segregation by countries and gender.

We enrolled 140 individuals with a confirmed genetic diagnosis of WHS between 2013 and 2023. Participants were recruited primarily from Spain (*n* = 75) and various Latin-American countries (*n* = 65), including Argentina, Mexico, Chile, Peru, Venezuela, Ecuador, and Colombia (see Figure 1). Some of the patients were previously described by us [9,27,28,29,30,31,32,33]. Figure 1 also shows the recruitment procedure of the patients over these years. Informed consent was obtained from the families, and all procedures complied with institutional ethical guidelines.

### 2.2. Clinical Data Collection

Clinical data were extracted from two standardized questionnaires and completed by referring physicians. Finally, they are validated through direct interviews with families and review of medical records. The data covered core WHS features, neurodevelopmental milestones, epilepsy characteristics, comorbidities, and social impacts. Two independent geneticists interviewed most of the parents to ensure consistency, minimize recall bias, and ensure standardized data from the questionnaires through manual review.

### 2.3. Genetic Characterization

All patients underwent high-resolution chromosomal microarray analysis using the Illumina Infinium CytoSNP-850k platform. Genomic deletions and duplications were annotated using GRCh37/hg19 coordinates and visualized via the University of California at Santa Cruz Genome Browser (http://genome.ucsc.edu/; (accessed on 16 January 2025)). Additional diagnostic techniques, including MLPA (MRC-Holland. Amsterdam, The Netherlands) and FISH, were used to confirm or further characterize chromosomal abnormalities when necessary.

### 2.4. Construction of the GFAP Score

The GFAP (Global Functional Assessment of the Patient) score was developed (using different variables from the questionnaires), as a continuous composite metric integrating four major clinical domains in the syndrome: (i) age-adjusted psychomotor milestones, (ii) epilepsy features, (iii) presence of comorbidities, and (iv) developmental aspects such as IUGR or hypotonia. Each item was scored based on clinical severity and weighted according to Human Phenotype ontology (HPO; https://hpo.jax.org/; (accessed on 20 January 2022)) term frequencies and clinical relevance. Finally, Principal Component Analysis (PCA) was applied to validate the GFAP construct, containing Kaiser–Meyer–Olkin’s measure and Bartlett’s test. The full scoring algorithm is detailed in Table S1.

### 2.5. Statistical Analyses

Descriptive statistics were used to characterize the cohort. The categorical variables were taken from our two questionnaires curated from medical records and were expressed as “1” (condition present at some point) or “0” (condition not present at any time). Pearson or Spearman correlations were used to assess relationships between GFAP and continuous or categorical variables, respectively. Comparisons between groups were made using Student’s *t*-test, Chi-square, or ANOVA with Bonferroni correction or T3- Dunnett post-hoc tests, where appropriate. The categorical variables were taken from our two questionnaires curated from medical records and were expressed as “1” (condition present at some point) or “0” (condition not present at any time). Hierarchical cluster analysis (Ward’s method) and discriminant analysis were used to assess the classification performance of GFAP. Statistical significance was defined as *p* < 0.05. Analyses were performed using SPSS version 28 (IBM Corporation, Chicago, IL, USA).

## 3. Results

### 3.1. Cohort Characteristics

The cohort comprised 140 individuals with a genetic diagnosis of WHS (64.3% female), with ages ranging from neonates to 39 years (mean 7.8; median 5 years). The median age at diagnosis was 11 months (mean 27.3 months). A complete list of clinical item frequencies and descriptive variables for the whole cohort is shown in Table S2 of Supplemental Data (Excel-based file; descriptive and frequency windows). Patients were predominantly from Spain and Latin America. Most individuals presented with global developmental delay and characteristic facial features, several comorbidities, and epilepsy. Feeding difficulties and organ anomalies were also common. Data such as neonatal data, dysmorphic traits, and comorbidities were presented in detail at Supplemental Data.

### 3.2. Genetic Findings

**Figure 2:**
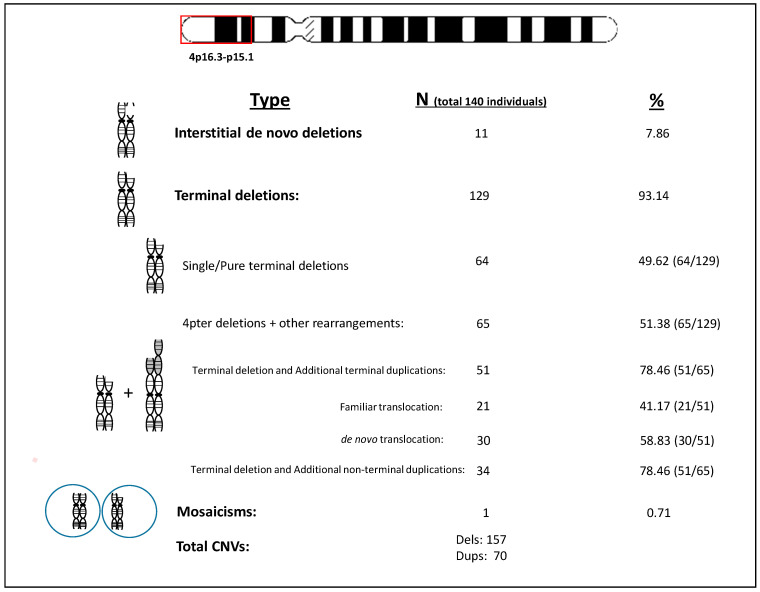
Type of genomic rearrangements found in the cohort.

Most patients (96%; 134/140) underwent SNP-array analysis. The 4-pter deletions were graphically represented using the UCSC Genome Browser database (2025 update), in Figure S1 of Supplemental Data. Genomic coordinates are also available in Table S2 of Supplemental Data (Coordinate window). Terminal deletions on 4p were the predominant rearrangement (87.1%; 122/140), with a subset of approximately 46% (65/140) of the cohort harboring complex or additional rearrangements, and 39.5% (51/140) exhibiting terminal duplications, suggestive of derivative chromosomes (see Figure 2). The cytogenetic data and analysis of the parents allowed us to establish the majority of them as *de novo* (30/51, around 58.82%) The average 4p deletion size was 9.12 ± 6.78 Mb (Table S2 of Supplemental Data; Coordinates window).

### 3.3. GFAP Score Distribution and Correlates

Table 1 shows the mean ± SD, median, and range values for GFAP and its intermediate “functional” components for this construct, in the whole cohort. The GFAP scores ranged from 64 to 410, with higher scores seeming to indicate a more severe functional impairment.

**Figure 3:**
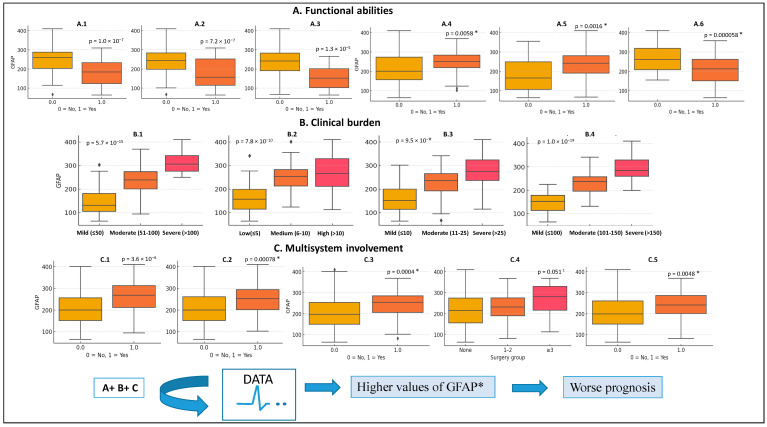
GFAP score (Arbitrary Units; AU) distribution in different functional abilities (**A**), clinical burden (**B**) or multisystem involvement (**C**) items recruited in our cohort. (**A1**), To be able to walk unaided; (**A2**), To be able to eat unaided; (**A3**), Ability to make sentences; (**A4**), Hearing problems. (**A5**), Non-sphincter control; (**A6**), To be able to sit unaided. (**B1**), Global epilepsy score (AU); (**B2**), Comorbidities score (AU); (**B3**), Developmental delay score corrected by age (AU); (**B4**), Developmental impact items score (AU). (**C1**), Cardiac anomalies; (**C2**), Nephro-urogenital anomalies; (**C3**), Ophthalmological anomalies; (**C4**), Number of surgeries; (**C5**), Recurrent respiratory infections. * *p* value statistically significant data using different statistical analysis approaches; t-Student, Chi-square, Mann–Whitney, Kruskal–Wallis.

Interestingly, significant positive correlations (using Pearson’s analysis) were observed between GFAP and deletion size, number of antiepileptic drugs used, and frequency of seizures and surgeries (*p* < 0.05). Negative correlations were found with control of epileptic crisis, gestational age, weight at birth, and cognitive/motor milestone achievements (*p* < 0.05; by Person’s analysis). In addition, a comparison between individuals with lower vs. higher GFAP scores at different functional and clinical items was shown in Figure 3A–C. Briefly, MRI anomalies (which are not shown in the figure), nephro-urological, cardiac anomalies among others functional (A), clinical burden (B), and multisystem (C) impairments were more frequent in high-GFAP individuals.

We further compared “functional” data, using the GFAP construct, with the different subpopulations and other different “queries”, such as gender, genetics, etc., in order to establish a potential “functional” variability among individuals with WHS. No significant differences in GFAP scores between subpopulations were found (Table S3 of Supplemental Data). Nevertheless, we did find significant GFAP differences between sexes. Indeed, males exhibited higher GFAP scores than females (*p* = 0.04; Student *t*-test, Table 2), consistent with a more severe functional profile, being associated with a higher number of surgeries, and larger size of the deletions, but inversely with neonatal parameters and or crisis control values (Student-*t* test *p* < 0.05). We also observed statistically significant differences among sexes comparing categorical variables (chi-square tests). Preferentially, in several motor or cognitive items, such as “*able to seat unaided*”, “*able to walk with help*”, “*able to walk unaided*”, “*non-sphincter control*” *or* “*communication with alternative method*” (*p* = 0.034, 0.029, 0.049, 0.038, 0.003, respectively), “*to be in monotherapy*” (*p* = 0.007), or different comorbidities items (“*C-gastrostomy*”, “*nephro-urogenital*”, *and* “*ophthalmology anomalies*”; *p* = 0.015; *p* = 0.0002; *p* = 0.024, respectively; Tau_ b Kendal). In all the cases, better numbers were observed in females, which were independent of the subpopulation used.

We also highlight that individuals with isolated (pure) 4p deletions had significantly higher GFAP scores than those with additional duplications (*p* = 0.03; Student-*t* test, Table 3). Notably, those differences are mainly established with some neonatal items. In fact, patients with additional duplications showed better neonatal outcomes (such as higher weight at birth and being born later in time; Student’s-test; *p* = 0.007 and 0.05, respectively) and less frequent intrauterine growth restriction (IUGR; *p* = 0.001, Fisher test, two sides). However, we have to note that those significant differences between the deletion size and to have or not to have an additional rearrangement could be a consequence of differences in deletion sizes (11.45 + 7.65 Mb, median 10.20 Mb in single 4p minus deletions versus 7.62 + 5.20 Mb, median 7.21 Mb in cases with an additional rearrangement; Student’s-test; *p* = 0.03).

Other comparisons among variables showing statistically significant differences (at GFAP) are shown in the Supplemental Data. Indeed, we compared individuals within whom non-febrile causes trigger seizures vs. fever as a trigger (Table S4 of Supplemental Data), or who had the ability to make sentences vs. not (Table S5 of Supplemental Data), or who had anomalies in MRI vs. not (Table S6 of Supplemental Data). Finally, families of individuals with higher GFAP scores were significantly more likely to report having quit jobs to provide the child care (*p* < 0.01), indicating that functional severity may impact caregiver burden (Table S7 of Supplemental Data).

### 3.4. Genotype/Phenotype Analysis

We further analyzed possible genotype/phenotype correlations based on the deletion size, and the existence of an additional rearrangement may modulate the GFAP score.

### 3.5. Ward’s Cluster Analysis Using the Size of the Deletion in the Whole Cohort

We performed Ward’s hierarchical cluster analysis by using “*deletion size*” as a variable (obtaining four clusters, see Table S8 of Supplemental Data) or by using two variables (“*deletion size*” and “*GFAP*”, Table 4) in order to verify how individuals may be grouped. Using the latest experimental approach, the individuals, at the end, were mainly grouped into two clusters (Akaike and BIC analysis in SPSS) as follows: cluster-A (deletion size, 18.98 ± 6.75 Mb; GFAP: 267.96 ± 53.12) and cluster-B (deletion size, 6.32 ± 3.40 Mb; GFAP: 215.87 ± 43.46). The number of patients in each group was 30 and 110 subjects, respectively. Table 4 shows the different items collected by both clusters (A and B) and is dissected by their frequencies.

Cluster-B, whose equivalence will be cluster-1 and -2 using the variable “deletion size” as a unique variable for clusterization (see Table S8 of Supplemental Data), showed better frequency/score values in different epilepsy items (up to 13, see “*global epilepsy*”), cognitive, and motor items than cluster-A. Cluster-B was also associated with a lower size of deletions and, thus, a lower score of GFAP, and its intermediates, aligning with a better prognosis than patients in cluster-A. Interestingly, we highlight that “*the ability to make sentences*” (expressive language) was associated preferentially with small deletions and, thus, with cluster-B (95%, 18/19). Something similar can be observed for individuals with the ability “*to say a few words*” (43/46; around 94%; Table 4) versus “*no words at all*”. Finally, according to the side effects, cluster-B individuals (smaller deletions) were diagnosed later in time (median: 12 months) than larger-size deletions (cluster-A’s individuals; median: 3 months. Student-*t* test *p* ≤ 0.05).

### 3.6. Unsupervised Hierarchical Clusterization and Discriminate Analysis

Unsupervised hierarchical clustering is one of the most common approaches to identify patterns in the data, without any prior knowledge about identifying characteristics, properties or classifications (unlabeled data) [30]. Clustering bietapic stepwise using all variables, but not GFAP or its intermediates, rendered two main groups of segregation that we called groups-1 and -2 (Table 5). Table 5 shows that group-1 segregated globally, with better numbers of the different variables studied. Indeed, Chi-square Student-t and Mann–Whitney tests showed significant differences between the two groups for most of the variables analyzed, including deletion size (10.85 ± 5.65 Mb for group-2, and 5.09 ± 3.68 Mb for group-1). Regarding GFAP, groups clearly also showed differences (261.80 ± 61.91 for group-2 and 176.19 ± 64.51 for group-1). Interestingly, there is a statistically significant association between sex and cluster membership. Indeed, males are overrepresented over females in group-2. The odds ratio indicates that a male is 4.5 times more likely to belong to group-2 than to group-1, compared to a female (*p* < 0.0031).

Discriminant analysis using GFAP as a unique variant classified 72.1% of patients correctly. Discriminant analysis is a classification challenge used to classify observations into non-overlapping groups, based on scores on one or more quantitative predictor variables. If we add “*deletion size*”, it is up to 77.2%. However, by applying just one of the GFAP’s intermediates, “*Development delay corrected by age*”, which reflects six motor and five cognitive milestone items corrected by age, we properly classified up to 89.0% of the original patients. In addition, adding the variable “*deletion size*” meant we got up to 93.4% of individuals. The threshold using “*Development delay corrected by age*” as a unique variable gave a value of 18.63. Below this score, individuals will segregate in group-2, with a better prognosis for the syndrome.

## 4. Discussion

Wolf–Hirschhorn syndrome (WHS) is a rare genetic disorder characterized by complex clinical manifestations and a severe prognosis. Although the general clinical aspects are frequently suggestive of the syndrome, the wide and varied spectrum of clinical manifestations in WHS may cause difficulties in diagnosis. This study presents the development and validation of the Global Functional Assessment of the Patient (GFAP), a clinician-reported outcome measure tailored specifically for individuals with WHS. By leveraging a large, clinically and genetically diverse cohort, we demonstrated that GFAP provides a reliable and quantifiable representation of functional severity in WHS. Our findings confirm that GFAP can capture key elements of disease burden, including neurodevelopmental delay, epilepsy, and comorbidities, while being sensitive to genetic variations such as deletion size and the presence of additional chromosomal rearrangements.

The GFAP score exhibited robust statistical associations with multiple clinical parameters, supporting that higher values of the GFAP score are associated with a worse prognosis (see Figure 3A–C). In addition, larger deletion sizes were significantly associated with worse functional outcomes (higher GFAPs), aligning with previous studies that suggest WHS follows a contiguous gene deletion syndrome, as it was previously indicated [31,32,33,34,35,36,37,38]. Interestingly, the presence of terminal duplications alongside 4p deletions appeared to mitigate some of the clinical severity, particularly in neonatal parameters such as birth weight and gestational age. These findings support the notion that additional genetic material (depending on the origin and type) may have a modulating effect on phenotype severity, a hypothesis also proposed in earlier investigations [39,40]. At this point, it is remarkable that only the adequate use of new genomic tools, such as high-resolution microarrays (CMA), may be used for a complete genetic diagnosis in WHS individuals. In fact, it is noteworthy that in many of the patients we also found an additional terminal duplication, among which those of the 8pter (*n* = 19) and 11pter (*n* = 7) chromosomes stood out. Thus, the complete genetic characterization in WHS individuals is important for not only diagnostic purposes, but also for prognosis, since given both the additional genetic alterations associated with 4p-deletions, and a higher/smaller size of it, can lead to a worse or a better functional prognosis, respectively. The observed attenuation of phenotype severity in individuals with additional duplications may reflect a genomic buffering effect, whereby duplicated segments exert compensatory transcriptional influences on pathways disrupted by the 4p deletion. This effect could be mediated by gene dosage interactions, chromatin reorganization, or epigenetic modulation. Alternatively, selective embryonic survival of more balanced rearrangements may contribute to this phenomenon.

Consistent with previous comments, WHS children have IUGR (IntraUterine Growth Restriction), which continues with failure to thrive during infancy [41,42]. However, many authors mentioned the putative relationship between IUGR and low weight at birth, but they do not correlate the weight at birth and gestational age of each patient. Our data showed a positive correlation between these two aspects, and also with the size of the deletions and, most interestingly, with the presence of those additional duplications, supporting that this fact responds to a genetic cause, supporting previous works [43].

Interestingly, sex-based differences were also observed, with males presenting with higher GFAP scores and more pronounced functional impairments. Although WHS is more frequently diagnosed in females, the increased burden in males suggests a potential sex-linked modifier effect or differences in healthcare access, reporting bias, or societal caregiving practices—areas that should have further investigation.

Cluster and discriminant analyses validated GFAP’s ability to stratify patients into clinically meaningful groups. Individuals in the lower-GFAP cluster demonstrated better cognitive and motor development, more effective seizure control, and fewer comorbidities. These insights offer clinicians a practical framework for anticipating patient trajectories and tailoring interventions. Notably, the GFAP subdomain related to age-corrected developmental milestones alone achieved near-equivalent classification performance, underscoring its value as a core prognostic indicator. Beyond its clinical utility, GFAP also revealed substantial social implications. Families of children with higher GFAP scores were more likely to experience significant life disruptions, such as leaving employment to provide care. This underscores the broader socioeconomic burden of WHS and the potential value of GFAP in identifying high-support families that may benefit from targeted resources and services. Although we did not observe significant differences in GFAP scores between Spanish and Latin-American cohorts, we acknowledge that underlying disparities in access to specialized care, early interventions, or educational support services may influence long-term outcomes beyond what is captured in the current analysis.

While our current data are cross-sectional, the GFAP may serve as a longitudinal outcome measure in natural history studies or therapeutic trials, capturing subtle shifts in developmental trajectories or responses to targeted interventions. Its use in future prospective cohorts may also allow evaluation of its sensitivity to clinical change, treatment response, and health service utilization. The framework underlying GFAP—integrating age-adjusted milestones, comorbidities, and seizure burden—may prove adaptable to other contiguous gene syndromes where functional heterogeneity poses similar clinical challenges. With appropriate recalibration, the GFAP concept may serve as a model for syndrome-specific ClinRO tools across the spectrum of rare neurodevelopmental disorders. In this sense, some previous experiences can be found in other rare diseases [44,45,46].

## 5. Future Address

Future studies may integrate GFAP scoring with detailed gene-level analyses to better understand how specific genes within the 4p16.3 region—such as *NSD2* (associated with developmental delay), *LETM1* (epilepsy), or *CPLX1* (synaptic function)—modulate individual trajectories. This could aid in refining prognostic models and targeting emerging precision therapies. A major strength of this study is the size and diversity of the cohort, which enhances the generalizability of our findings. However, limitations include the cross-sectional nature of data and the reliance on retrospective questionnaire-based assessments. Future longitudinal studies are needed to assess GFAP’s sensitivity to change over time and its responsiveness to interventions.

In future work, incorporating age-stratified analyses may help delineate how functional trajectories evolve with age and identify potential developmental plateaus or critical windows. Lastly, internal cross-validation techniques could be applied to confirm the robustness of the GFAP model and its subdomains across different clinical subgroups.

## 6. Conclusions

This study introduces and validates the Global Functional Assessment of the Patient (GFAP) as a potential first clinician-reported outcome assessment specifically designed for individuals with Wolf–Hirschhorn syndrome. The GFAP score represents a novel, WHS-specific ClinRO capable of stratifying patients by functional severity and supporting clinical decision-making. Its integration into both clinical practice and research protocols may improve care planning and enable more precise evaluation of future therapeutic strategies. Importantly, GFAP demonstrates the capacity to distinguish between clinically meaningful subgroups of WHS patients, facilitating tailored care approaches and offering valuable insights into the impact of chromosomal complexity on disease expression. The integration of GFAP into clinical practice could enhance patient monitoring, support family counseling, and guide future interventional trials. Practical implementation could involve embedding the GFAP score into electronic health records for routine use during clinical assessments, and its application as an endpoint in prospective natural history or treatment studies.

## Figures and Tables

**Table 1:** Median and mean values for this GFAP and its intermediate components in the whole cohort.

	Mean	Median	Range
GFAP	227.23 ± 75.64	235.00	64–410
i.- Developmental delay milestones corrected by age	19.78 ± 11.97	16.00	2–45
ii.- Comorbidities	8.15 ± 5.26	7.00	0–45
iii.- Several items affecting developmental aspects	130.40 ± 43.38	131.50	30–230
iv.- Global epilepsy	68.45 ± 31.89	67.00	0–170

GFAP, Global Functional Assessment of the Patient.

**Table 2:** Median and mean values for this GFAP and its intermediate components by gender.

Gender	Males (*n* = 44)			Females (*n* = 95)		
	Range	Median	Mean	Range	Median	Mean
GFAP	64–410	254	248.09 ± 88.09 *	82–359	219	217.25 ± 67.20
i.- Developmental delay items corrected by age	2–40	27.50	22.50 ± 11.87 ^t^	2–45	15	18.48 ± 11.86
ii.- Comorbidities	1–21	9.0	9.32 ± 3.99 ^t^	0–45	8.0	7.59 ± 5.7
iii.- Different items affecting developmental aspects	30–230	150	141.16 ± 47.73 *	35–210	128.0	125.25 ± 40.39
iv.- Global epilepsy	0–170	77	75.95 ± 39.21	0–135	65	64.86 ± 27.24
Motor Milestones (*motor delay*) (up to 6)	0–6	3	2.73 ± 1.89 *	0–6	4	3.72 ± 2.18
Cognitive milestones (*cognitive delay*) (up to 5)	0–5	2	2.30 ± 1.27 *	0–5	3	2.75 ± 1.29

^t^, tendency = 0.05–0.065; * *p* ≤ 0.05, *t*-Student Test. GFAP, global functional assessment of the patient.

**Table 3:** Median and mean values for this GFAP and its intermediate components by genetic findings.

Genetics	4p Minus Single Deletions (*n* = 63)			4p Minus Deletions + Additional Rearrangement (*n* = 69)		
	Range	Median	Mean	Range	Median	Mean
GFAP	102–410	256.50	246.07 ± 71.36	82–369	221	218.74 ± 71.35 *
i.- Developmental delay items corrected by age	2–45	21.50	22.27 ± 12.08	2–21	15	17.78 ± 11.39 *
ii.- Comorbidities	2–45	8.0	9.52 ± 6.31	0–45	7.0	7.33 ± 4.01
iii.- Several items affecting developmental aspects	30–230	150.50	141.02 ± 40.23	30–230	128.0	124.28 ± 43.11 *
iv.- Global epilepsy	0–170	68	73.58 ± 31.90	20–135	65	67.64 ± 28.51
Motor Milestones (up to 6)	0–6	3	2.92 ± 2.03	0–6	4	3.74 ± 1.95 *
Cognitive milestones (up to 5)	0–5	2	2.50 ± 1.28	0–5	2	2.62 ± 1.26

* *p* ≤ 0.05, *t*-Student Test. GFAP, global functional assessment of the patient.

**Table 4:** Ward’s hierarchical cluster variable frequency analysis using deletion size and GFAP values.

Variable	Cluster A	Cluster B
Gender (Female/Male)	18F/12M (1.50:1)	77F/33M (2.33:1)
size of deletion (Mb)	18.98 ± 6.75 (15.98) range 12.00–41.50	6.37 ± 3.45 (6.26) range 0.01–15.46
Subpop Sph/Lat	17/13 (1.31:1)	57/53 (1.07:1)
Age at evaluation (years)	5.11 ± 5.76 (3.12) range 0.01–34.04	8.53 ± 8.44 (5.60) range 0.01–39.04
Age at diagnosis (months)	12.97 ± 27.87 (3) range 0.01–144	31.11 ± 56.92 (12) range 0.1–384
Additional duplications	Not, 23/Yes, 7 (30.43%)	Not, 55/Yes, 55 (50%)
GFAP score (AU)	267.96 ± 53.12 (261) range 164–369	215. 87 ± 77.07 (220) range 64–346
Weighted Psychomotor delay milestones corrected by age	28.19 ± 10.32 (30) range 2–45	18.83 ± 11.56 (17) range 2–38
Comorbidities	9.70 ± 5.26 (9) range 2–23	7.58 ± 4.13 (7) range 0–21
DD affecting items	148.67 ± 37.96 (151) range 80–150	125.87 ± 43.46 (126) range 30–190
Global Epilepsy items	81.41 ± 26.23 (92) range 32–98	64.96 ± 37.72 (65) range 0–150
Prenatal/Neonatal		
IUGR	29/29 (100%)	101/109 (92.70%)
Medro faillure	23/27 (85.20%)	101/109 (92.70%)
Gestational week	35.65 ± 4.37 (37) range 17–42	36.87 ± 2.44 (37) range 28–41
Weight at birth (gr)	1830.19 ± 414.36 (1800) range 440–2700	2049.14 ± 498.13 (2010) range 840–3700
height at birth (cm)	42.48 ± 3.33 (43) range 29.5–46	44.09 ± 3.52 (44) range 33–52
OFC at birth (cm)	30.76 ± 3.15 (31) range 19.5–39	31.46 ± 0.26 (31) range 21–39
EPILEPSY		
Seizures	27/28 (96.50%)	99/109 (90.80%)
Age of seizures (months)	7.72 ± 4.49 (8) range 0–18	10.26 ± 7.03 (9) range 0.01–36
Seizures w fever	16/27 (59.30%)	79/107 (73.80%)
Seizures w/o fever	20/27 (74.10%)	61/107 (57.0%)
Status	18/27 (66.70%)	62/107 (57.90%)
Number status	3.56 ± 6.07 (2) range 0–30	2.93 ± 6.56 (1) range 0–55
Status to ICU	12/27 (44.40%)	46/107 (43.0%)
AEDs	25/27 (92.600%)	90/107 (84.10%)
Number of AEDs	2.15 ± 1.13 (2) range 0–5	2.16 ± 1.52 (2) range 0–6
Monotherapy	12/27 (48.10%)	50/108 (46.29%)
Max number of AEDs used simultaneously.	1.81 ± 1.0 (2) range 0–5	1.54 ± 0.90 (1) range 0–4
Took drug for epilepsy not now	3/27 (11.10%)	17/108 (15.74%)
Crisis control (1 to 6)	2.52 ± 1.31 (2) range 0–5	3.91 ± 1.58 (5) range 1–6
MOTOR		
Able to support head	18/27 (66.70%)	104/110 (94.54%)
Able to seat	14/27 (51.90%)	89/110 (80.90%)
Able to seat unaided	9/27 (33.30%)	83/110 (75.45%)
Able to walk with help	7/27 (25.90%)	71/110 (64.54%)
Able to walk unaided	13/27 (48.10%)	48/110 (43.64%)
Able to eat unaided	2/27 (7.40%)	36/110 (32.72%)
COGNITIVE		
Non-sphincter control	24/27 (88.90%)	82/110 (74.54%)
Able to communicate with environment	24/27 (88.90%)	100/110 (90.90%)
Communication with alternative tools	14/27 (51.90%)	81/110 (73.66%)
Able to say some words	3/27 (11.10%)	44/110 (40.0%)
Able to make short sentences	1/27 (3.70%)	18/110 (16.36%)
Motor milestones total	1.75 ± 1.58 (2) range 0–6	3.84 ± 1.91 (4) range 0–6
Cognitive milestones total	2.11 ± 1.22 (2) range 0–5	2.74 ± 1.29 (3) range 0–5
COMORBIDITY		
C-gastrostomy	3/27 (11.10%)	12/110 (10.90%)
Cardiovascular problems	13/27 (48.10%)	48/110 (43.63%)
Nephro-urogenital anomalies	19/27 (70.40%)	53/110 (48.18%)
Ophthalmological problems	20/27 (74.10%)	58/110 (52.72%)
Auditive problems	12/27 (44.40%)	44/110 (40.0%)
Recurrent air tract infections	17/27 (63.0%)	62/110 (56.36%)
Brain anomalies by MRI	15/21 (71.42%)	58/110 (52.72%)
Number of Surgeries	1.22 (1) range 0–6	0.93 (1) range 0–7
SOCIAL		
A family member quit job to care	15/27 (55.60%)	8/22 (36.36%)

Mb, megabases. AU, arbitrary units. DD, developmental delay. Sph/Lat, Spanish/Latin-American. IUGR, intrauterine growth restriction. ICU, intensive care unit. OFC, head circumference. AEDs, anti-epileptic drugs. GFAP, global functional assessment of the patient; MRI, magnetic resonance imaging.

**Table 5:** Unsupervised hierarchical cluster segregation in the whole cohort. Summary of differences between group-1 and group-2.

Variable	Group-1 (Mean)	Group-2 (Mean)	*p*-Value	More Affected Group
Gender	Male, 17.20%; female, 82.80%	Male, 48.50%; female, 52.50%	0.0031	
Deletion size (Mb)	5.09 ± 3.68	10.85 ± 5.65 ▲	0.00001	group-2
GFAP score (AU)	206.24	342.14 ▲	<0.00001	group-2
Develomental delay corrected by age	17.63	31.52 ▲	<0.00001	group-2
Global epilepsy	61.97	103.90 ▲	<0.00001	group-2
Comorbidities	7.30	12.81 ▲	<0.00001	group-2
Alterations affecting development	119.82	188.33 ▲	<0.00001	group-2
Motor delay	3.67 ▲	1.90	0.0002	group-2
Cognitive delay	2.76 ▲	1.76	0.0011	group-2
Number of AEDS	1.70 ± 1.44	2.46 ± 1.37 ▲	0.003	group-2
Cardiovascular anomalies	0.37	0.86 ▲	0.0001	group-2
Nephro-urological anomalies	0.50	0.71 ▲	0.0014; Odds ratio = 0.3	group-2
Ophthalmological anomalies	0.54	0.71 ▲	0.05; Odds ratio = 0.47	group-2
Recurrent respiratory infections	0.55	0.71 ▲	0.05; Odds ratio = 0.47	group-2
Non-sphincter control	0.67	0.87 ▲	<0.001; Odds ratio = 0.074	group-2
MRI anomalies	0.53	0.70 ▲	0.012	group-2
IUGR	0.93	1.00 ▲	0.06^t^; Odds ratio = 0.15	group-2
Seizures	0.91	0.95 ▲	0.016; Odds ratio = 0.11	group-2
Status	0.57	0.71 ▲	0.0003; Odds ratio = 0.249	group-2
Number of Status	2.97	3.48 ▲	0.019	group 2
Able to support head	0.92 ▲	0.71	0.00028; Odds ratio = infinite	group-2
Able to seat unaided	0.73 ▲	0.33	0.0010	group-2
Able to communicate to the environment	0.75 ▲	0.38	0.0009	group-2
Able to walk unaided	0.43 ▲	0.05	0.0022	group-2
To be able to say words	0.38 ▲	0.10	0.011	group-2
To be able to make sentences	0.17 ▲	0.001	0.0012	group-2
Weight at birth	2,120 ± 536 ▲	1,930 ± 44.2	0.026	group-2
OFC at birth	32.17 ± 2.71 ▲	30.78 ± 2.73	0.006	group-2
Age at diagnosis (months)	41.04 ± 61.27 ▲	18.91 ± 44.83	0.006	group-2
Age at evaluation (years)	11.32 ± 9.62 ▲	5.69 ± 5.92	0.00001	group-2
Familiar has to quit her/his job to care	0.54	0.76 ▲	0.064 ^t^; Odds ratio = 0.5	group-2

Represented only differences among variables that showed statistical significance via either t-student, Mann–Whitney or chi-square tests. ^t^, means tendency. ▲, means higher value. IUGR. Intrauterine growth restriction. OFC. Head circumference. AU, arbitrary units. AEDS. Anti-epileptic drugs. Mb, megabases.

## Data Availability

We deposited data from SNP-arrays in the DECIPHER database (v11.33) repository (#562946 to 563086; IMMGWHS1-140). Additional data is available in the Supplemental Data Section.

## Supplementary Materials

The following supporting information can be downloaded at: https://www.mdpi.com/article/10.3390/genes16070820/s1, Figure S1: SNP array results for the whole cohort; Table S1: Items for constructing GFAP; Table S2: Descriptive and frequency data for the whole cohort; Table S3: Median and Mean Values for this GFAP and its intermediate components in the subpopulations. Table S4: Median and Mean Values for this GFAP and its intermediate components by individuals with seizures without fever vs not; Table S5: Median and Mean Values for this GFAP and its intermediate components by individuals with the ability to make sentences vs not; Table S6: Median and Mean Values for this GFAP and its intermediate components by individuals with MRI anomalies vs not.; Table S7: Median and Mean Values for this GFAP and its intermediate components by individuals who quit the job vs not; Table S8: Ward’s hierarchical cluster analysis using “deletion size” as unique variable; Table S9: Descriptive and frequency data for Subpopulations; Auxiliary Methods; Additional Results.
