# Supplementary material for: Clinician-Based Functional Scoring and Genomic Insights for Prognostic Stratification in Wolf–Hirschhorn Syndrome
Source: Genes (Basel). 2025 Jul 12;16(7):820. doi: 10.3390/genes16070820 (PMC12294571; doi:10.3390/genes16070820)
Supplement: Supplementary file 1 [file genes-16-00820-s001.zip › finalsuppl.pdf]

Supplementary data.

Article title: Clinician-Based Functional Scoring and Genomic Insights for Prognostic Stratification in Wolf-Hirschhorn Syndrome

Journal name: Genes

Authors: Nevado, J et al.,
Affiliation and e-mail address of the corresponding autor: Julián Nevado, BSc, Ph.D, MBA. Instituto de Genética Médica y Molecular (INGEMM), IdiPAZ; Hospital Universitario la Paz, Paseo de la Castellana 261, Madrid, 28046, Spain.
Mail to: jnevado@salud.madrid.org

AUXILIARY METHODS.

1.- Karyotyping and FISH

Cytogenetic analyses were performed on GTG-banded metaphases at a resolution of about 550 bands according to standard laboratory protocol using Chromosome Kit-P (Euroclone, Siziano PV, Italy). FISH was performed according to standard laboratory protocols using commercial subtelomeric 4-pter probes (available upon request).

2.- MLPA probe kits

We used several commercial MLPA (MRC-Holland, Amsterdam, The Netherlands) kits in this study. For patients referred to rule-out subtelomeric rearrangements in the first years of the study, we applied MLPA kits P036 and P070. DNA samples of all the 4p-ter deletions were further characterized by applying the specific 4p-ter MLPA P096 probe mix. Data analyses were performed according to the protocols supplied by the provider. The ratio of each peak's relative probe area was then compared versus a DNA control sample (Promega, UK), using Coffalysser v.9.4 (MRC-Holland; The Netherlands).

3-SNP-array Analysis

A genome-wide scan of 850,000 tag SNPs (Illumina Infinium CytoSNP-850k v1.1 and v1.2 BeadChip) was applied (Illumina, San Diego, CA) (Figure 1 Supplemental data). All genomic coordinates were established according to the 2009 human genome build 19 (GRCh37/NCBI build 37.1)

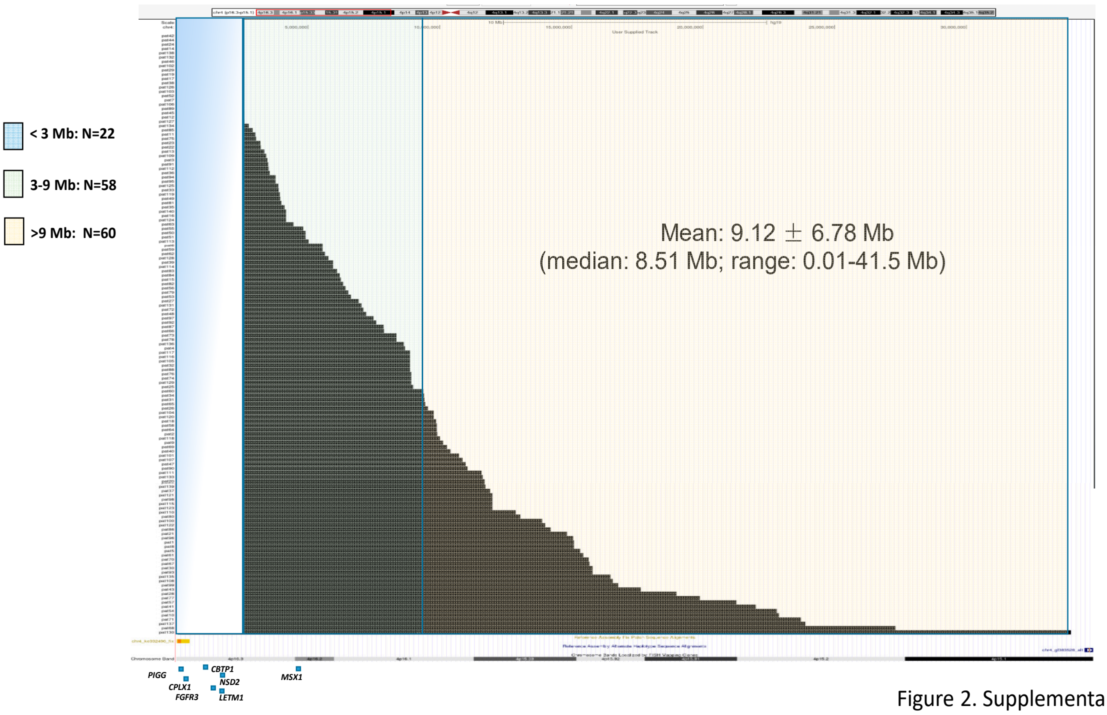

Figure 2. Supplemental data

Figure S1. SNP array results for the whole cohort. Deletion sizes were plotted on the genome browser using the University of California at Santa Cruz Genome Browser (http://genome.ucsc.edu/). Individuals are classified by size of the 4p deletions into three groups; < 3 Mb in blue, 3 to 9 Mb in light green [illegible] cream.

4-Global Functional Assessment of the Patient (GFAP) in our cohort

We estimated individual "functional" assessment in our cohort by using different variables from the questionnaires weighed them by HPO frequencies in a numerical scale of the four main clinical items in the syndrome, under our clinical experience. Final patient assessment (GFAP) was developed by the sum of items "I" to "IV" (Table 1 of Supplemental data).

Table S1. Items for constructing GFAP.

**VARIABLES**	**to have**	**not to have**
**i) Psychomotor milestones corrected by age:**		
**Motor**		
cephalic support	0 points	20 points
able to be seated	0 points	20 points
able to be seated unaided	0 points	20 points
able to walk	0 points	20 points
able to walk unaided	0 points	20 points
able to eat unaided	0 points	20 points

Cognitive		
use diapers	20 points	0 points
no words at all	20 points	0 points
use alternative communication tools	-5points	0 points
a few words	-10 points	0 points
language with short sentences	-20 points	0 points
**ii) Comorbidity**		
Auditive problems	1 points	0 points
optalmologic alterations	1 points	0 points
recurrent respiratory infections	1 points	0 points
cardiac problems	1 points	0 points
Feed difficulties	20 points	0 points
gastroesophagic reflux	20 points	0 points
Scoliosis	20 points	0 points
Hypotonia	20 points	0 points
Seizures	20 points	0 points
Nephrourogenital anomalies	1 points	0 points
skin problems	1 points	0 points
normal EEG	0 points	20 points
normal Electro cardiogram	0 points	20 points
normal metabolic screening	0 points	20 points
Surgeries	2pts/each	0 points
Others (each one)	10 points	0 points
**iii) Developing delay affecting items**		
MRI anomailies	20 points	0 points
hypotonia	20 points	0 points
seizures	20 points	0 points
conductual problems	10 points	0 points
IUGR	20 points	0 points
Medro faillure	20 points	0 points

gestational week under the mean value	5-20 points	up 0 points
weight at birth under the mean value	5-20 points	up 0 points
height at birth under the mean value	5-20 points	up 0 points
OFC at birth under the mean value	5-20 points	up 0 points
developmental delay	20 points	0 points
ID	20 points	0 points
Feed difficulties	20 points	0 points
Microcephaly	20 points	0 points
sleeping problems	20 points	0 points
**iv) Global epilepsy items**		
seizures	20 points	0 points
Age of seizures: < 6 months<br>*6-12 months*<br>*> 12 months-4 years*<br>*> 4 years*	20 points<br>10 points<br>5 points<br>0 points	
Seizures with fever	10 points	
Seizures without fever	20 points	
Status	20 points	0 points
Number status: 1-5<br>*>5*	10 points<br>20 points	
Status to ICU	20 points	0 points
Monotherapy	-20 points	0 points
Took drugs for epilepsy not now	-20 points	0 points
Number of AEDs: ≤2<br>*>3*	10 points<br>20 points	
Max number of AEDs taken simultaneously:<br>*2*<br>*>2*	 10 points<br>20 points	
Control of crisis: 1-3<br>*4*	20 points<br>10 points	

5	-10 points	
6	No epilepsy	

IUGR, intrauterine growth restriction. ID, intellectual disability. OFC, head circumference. ICU, intensive care unit. AEDs, anti-epileptic drugs.

ADDITIONAL RESULTS.

1. Description of the cohort.

The cohort (140 individuals) has a mean age of 7.82 years (median 5 years) with a range of age between one day and 39 years-old. The mean age at diagnosis was 27.30 months (median 11 months). A complete list of clinical item frequencies and descriptive variables for the whole cohort is shown in Table S2 of Supplemental data (Excel-based file; descriptive and frequency windows).

1.1. Neonatal data

Births were mostly at pre-term stage, with an average gestational age of 36.63±2.95 weeks (median 37 weeks) and ranged between 17 and 42 weeks. The average birth of weight, height, and cranial perimeter were low values for their gestational age [41-42]. Mostly of the individuals (94.20%, 131/139) showed a delay in intrauterine growth (IUGR) during pregnancy monitoring, and which continues with some delaying aspects in the postnatal growth in 84.55% of individuals (115/136). Although many of individuals experimented feeding problems at birth, only around 11.76% (16/136) of the cases have been subjected to G-tube gastrostomy.

1.2. Dysmorphic traits and physical aspects.

Most of the patients from this cohort showed the classic facial gestalt of WHS, consisting in dysmorphic features resembling a Greek warrior helmet-shaped face [37]. Only interstitial cases (8 patients), but especially three of them, did not show any dysmorphic features, according to have a small loss of genetic material beyond the critical region of this syndrome (Data not shown). It has been previously suggested that, this phenotype becomes less evident at older the age [32, 33], and is usually related to the size of the deletion [32, 33]. We fine revised by scoring, this facial appearance in 19 individuals from the cohort by two clinical expert dysmorphologists. We have found that, in one of them the improvement is evident, but in other four a worsening have been observed, and in the rest (14 cases), there are no obvious changes (data not shown).

1.3. Comorbidity.

Regarding surgeries as a mark of comorbidity, almost 55% (74/135) of the patients experimented with at least one, ranging from one to seven. Remarkably, MRI studies reveal that approximately 74% (74/100) of the patients have any brain-image alteration. Often, they are nonspecific findings or lesions (such as cortical- or subcortical-atrophy, abnormalities of the corpus callosum, ranging from thinness to agenesis, lesions of the white substance or prominence of ventricles without suggestive data of active hydrocephalus). Interestingly, around 75% (100/133) of WHS individuals have at least one MRI done. Others comorbidities; subjects experimented heart anomalies, hearing problems, nephro-urological problems, ophthalmological anomalies, or recurrent upper way tract infections vary from 44 to 57%, (see Table S2 of Supplemental Data).

Most of the individuals (≥80%) have cognitive delay, which has been categorized in moderate to severe based on the number of neurodevelopmental milestones achieved based on their age (scale up to 2.60 of 5; median of 3; where 5, means normal milestones; Table 2 of Supplemental Data). Unlikely to observed on cognitive delay milestones, around 55% of individuals are considered to have a light or moderate motor delay (scale up to 3.40 of 6; median

of 4; where 6, means normal milestones; Table 2 of Supplemental data). From a social point of view, approximately 81.5% (109/134) of families had bad sensations regarding their child's health before they were diagnosed. In fact, more than half of the parents (at least one) have quitted their jobs to care for WHS individuals (77/135, 57%), mostly the mothers.

1.4. Epilepsy.

Epilepsy is one of the core clinical items in WHS, which appears in 91.90% (126/140) of cases of our cohort, with an average age at the debut of 9.75±6.66 months (median of 9 months), with 78/126 (63.41%) of the cases with an age of onset between 6-12 months. Regarding characteristics of seizures, it should be noted that 70.90% (96/126) of the children showed fever-related seizures, and 60.40% (82/126) of them also have seizures without fever (e.g. triggering by some infections or stress conditions). At the time of evaluation more than half of children (around 60%; 80/126) had had at least one epileptic status episode, resulting in the average patient status of 3, where 72.50% (58/80) needed get into the ICU of the Hospital (Table 2 Supplemental data, see Excel). Overall, the seizures observed were multi-type (data not shown), with an average number of drugs tested per patient of 2.16±1.44 (median of 2). Interestingly, around half of the individuals with epilepsy were in monotherapy (64/126; 50.79%). We highlight that, half of the children have been at least a year of crisis-free at the time of this evaluation (63/126), although around 15% (19/126) of the individuals still describe monthly seizures despite of the treatment used (Table 2 of Supplemental data).

3.-Individual Global Functional Assessment of the Patient (GFAP)

3. 1. Comparisons by subpopulations

We compared both subpopulations among them (Table S3 Supplemental data). No great differences can be observed between these two populations.

Table S3. Median and Mean Values for this GFAP and its intermediate components in the subpopulations.

Subpopulations	Spanish (n=75)			Latin-American (n=64)		
	Range	Median	Mean	Range	Median	Mean
**GFAP**	**64-369**	**231**	**223.35±77.15**	**67-410**	**239**	**231.46±74.32**
**i.- Psychomotor milestones corrected by age**	2-40	18.0	18.86±11.68	2-45	15	20.78±12.32
**ii.- Comorbidities**	0-45	7.0	7.30±5.56	2-22	8.0	9.08±4.79
**iii.- Different items affecting developmental aspects**	30-230	137	133.39±46.85	50-210	130.0	127.12±39.33
**iv.- Global epilepsy**	0-135	64	64.58±31.63	0-170	72.0	72.68±31.89

GFAP, global functional assessment of the patient.

3. 2. Comparisons by reasons triggering seizures

Fever is one of the main causes that is often thought of triggering seizures, but it seemed to be

associated to less intense/severe crisis episodes. We compared whether seizures with or without fever may have different contribution in the GFAP values. We highlight the data regarding only individuals who have seizures without fever. The GFAP, its intermediates, and other such as motor and cognitive milestones, control of epileptic crisis, number of status-epileptic episodes, etc, showed significant differences between the groups (see Table 4 Supplemental data). Most of the GFAP data showed "functional" worse data in individuals which suffered seizures without fever than other individuals showing epilepsy only with fever or in a combined way, as a causes of triggering seizures. Chi square analysis also reflected worse data (such as status that involves visiting ICU, different types of seizures, different treatments, to use diapers, say words or elaborate sentences) for those they have only seizures without fever vs. those with fever. However, in the other way around, some motor issues seemed to be better in those individuals with seizures with no fever (able to walk unaided, able to eat unaided) vs individuals with fever-induced episodes. Finally, the group of patients with seizures and no-fever was also associated with those with higher percentage of CNS (central nervous system) malformations, intellectual disabilities and C-gastrostomy, higher number of status, higher total number of AEDs taken simultaneously, and lower values of crisis control. Individuals with fever-triggered seizures did not show any statistically significance difference, at any variable (by Chi-square or Student-t tests, data not shown).

Table S4. Median and Mean Values for this GFAP and its intermediate components by individuals with seizures without fever vs not.

Individuals with seizures without fever	YES<br>Del size 9.77±5.40 Mb*<br>N=81	NOT<br>Del size 7.12±5.7 Mb<br>N=53
	Mean	Mean
**GFAP**	**257.94±65.97***	184.74±65.98
i.- Developmental delay items corrected by age	**22.38±11.30***	15.70±11.90
ii.- Comorbidities	No differences	No differences
iii.- Several items affecting developmental aspects	**141.21±40.90***	115.49±42.0
iv.- Global epilepsy	**84.19±26.35***	46.98±23.0
**Motor Milestones (up to 6) (*motor delay*)**	**3.06±1.96***	3.92±1.92
**Cognitive milestones (up to 5) (cognitive delay)**	**2.31±1.22***	3.04±1.25

** p≤0.05, t-Student Test. GFAP, global functional assessment of the patient*

3.3. Comparisons by who showed the ability to make sentences

We also compared whether the ability to make sentences may also be counted in the GFAP score. We globally observed statistically significant differences at the GFAP (and its intermediates; Table 5 Supplemental data), between the individuals with this ability and who were not. GFAP data were better in the individuals showing this ability. This group, as we expected, is also associated with better "functional" numbers in cognitive and motor issues, crisis control (Yes; 4.05, Not; 2.63 over 6), deletion size, number of surgeries, maximum number of antiepileptic drugs taken, and age at evaluation (Yes; 9.23 yrs., Not; 4.99 yrs.); and diagnosis (Yes; 33.63 months, Not; 15.40 months). Chi square analysis also reflected statistically significance (p=0.035) and better numbers for those they are able to make sentences on the following variables; AEDs taken, to have seizures, motor and cognitive milestones, intellectual disability, nephron-urogenital disease, seizures with no fever, CNS malformations, communication with the environmental skills, ability to say words.

Table S5. Median and Mean Values for this GFAP and its intermediate components by individuals with the ability to make sentences vs not.

Individuals with ability to make sentences	YES<br>Del size 4.06±4.46 Mb*<br>N=19	NOT<br>Del size 9.40±5.56 Mb<br>N=115
	Mean	Mean
**GFAP**	155.16±59.04*	**238.93±71.62**
i.- Developmental delay items corrected by age	4.63±5.71*	**22.40±10.86**
ii.- Comorbidities	4.79±2.66*	**8.69±5.38**
iii.- Items affecting developmental aspects	104.37±43.86*	**134.62±41.98**
iv.- Global epilepsy	75.16±22.35*	**81.6±23.4**
**Motor Milestones (up to 6) (*motor delay*)**	5.74±0.73*	**3.02±1.91**
**Cognitive milestones (up to 5) (cognitive delay)**	4.63±5.72*	**2.27±1.04**

** p≤0.05, t-Student Test. GFAP, global functional assessment of the patient*

3.4. Comparisons by who showed anomalies in the MRI.

We also compared patients who showed MRI anomalies versus who did not. We globally observed significant differences between the individuals with anomalies, at the GFAP data (and its intermediates; Table 6 Supplemental data), as well as motor and cognitive milestones. Better numbers were observed in individuals without anomalies in the MRI.

Table S6. Median and Mean Values for this GFAP and its intermediate components by individuals with MRI anomalies vs not.

Individuals with anomalies in CNS by MRI	YES<br>Del size 9.34±5.45 Mb<br>N=73	NOT<br>Del size 7.70±5.56 Mb<br>N=59
	Mean	Mean
**GFAP**	**245.55±63.17***	**208.10±60.07**
i.- Developmental delay items corrected by age	No differences	No differences
ii.- Comorbidities	**9.67±1.70***	**6.51±1.80**
iii.- Items affecting developmental aspects	**138.90±41.50***	122.50±43.2
iv.- Global epilepsy	**75.16±22.35***	61.22±23.2
**Motor Milestones (up to 6) (*motor delay*)**	**2.95±1.60***	3.93±1.20
**Cognitive milestones (up to 5) (cognitive delay)**	**2.30±1.56***	2.93±1.10

** p≤0.05, t-Student Test. GFAP, global functional assessment of the patient*

3.4. By families that they do quit a job to take care of WHS individuals.

We also check whether or not the worse functional numbers may also affect putative quality life of the families, we measure the option to quit a job to care WHS children. Again, GFAP and its intermediates showed significant differences among parent who do quit their jobs ($p \leq 0.05$, Student-t test). Better number of GFAP was observed in parents that did not quit the jobs (Table 7 of Supplemental data).

Table S7. Median and Mean Values for this GFAP and its intermediate components by individuals who quit the job vs not.

Families that they quit a job	YES<br>Del size 9.15±5.93 Mb<br>N=77	NOT<br>Del size 7.89±5.52 Mb<br>N=58
	Mean	Mean
**GFAP**	**244.91±72.25***	205.00±74.69

i.- Developmental delay items corrected by age<br>ii.- Comorbidities<br>iii.- Items affecting developmental aspects<br>iv.- Global epilepsy	**21.49±12.06***<br>No differences<br>**139.16±41.48***<br>**74.64±31.01***	17.24±11.44<br>No differences<br>118.86±43.84<br>61.41±30.69
**Motor Milestones (up to 6) (*motor delay*)**	No differences	No differences
**Cognitive milestones (up to 5) (cognitive delay)**	**2.43±1.28***	2.86±1.27

** p≤0.05, t-Student Test. GFAP, global functional assessment of the patient*

4. Genotype-Phenotype analysis

4.1. Ward´s Cluster Analysis by the size of the deletion in the whole cohort

Ward's hierarchical cluster analysis by deletion size as unique variable is shown in Table 8 of Supplemental data.

Table S8. Ward´s hierarchical cluster analysis using "deletion size" as unique variable.

Variable	Cluster 1	Cluster 2	Cluster 3	Cluster 4
**Gender (Female/Male)**	14F/7M (2:1)	**33F/8M (4.12:1)**	36F/17M(2.11:1)	9F/12M (0.75:1)
**size of deletion (Mb)**	3.32±2.49(2.9) range 0.01-9.50	7.47±5.36(5.55) range 1.30 -23.73	10.01±6.12(8.92) range 1.39-27.30	**12.52±5.27(12) range 5.94-23.90**
**Subpop Sph/Lat**	12/9	22/19	25/28	12/9
**Age at evaluation (years)**	**13.17±10.68 (9.30) range 2.15-31.24**	6.07±7.24 (3.97) range 0.01-34.04	8.42±7.42 (5.96) range 1.43-33.93	5.05±5.60 (3.10) range 0.03-24
**Age at diagnosis (months)**	**53.29±72.73 (39) range 0.1-323**	20.15±33.93 (10) range 0.001-194	25.75±58.31 (10) range 0.01-384	23.17±41.83 (10) range 0.1-144
**Additional duplications**	09/21(42.85%)	**21/41(51.21%)**	23/53 (43.39%)	8/21 (38.09%)
**GFAP score (AU)**	108,24±20,57(113) range 64-136	185.39±21,57(188) range 149-221	261,21±20,65(260) range 225-301	**342.14±27,44(336) range 303-410**

Weighted Psychomotor delay items	7.86±8.54 (5)<br>range 2-35	16.61±10.87(15)<br>range 2-40	22.30±10.34(18)<br>range 2-45	**31.52±6.85(33)**<br>**range 15-45**
Comorbidities	4.05±2.64(4)<br>range 0-11	7.46±4.88(7)<br>range 2-22	8.45±3.13(8)<br>range 4-19	**12.81±8.10(11)**<br>**range 4-45**
DD affecting items by age	73.10±26.11(75)<br>range 30-142	107.71±27.53(99)<br>range 65-171	147.70±22.05(151)<br>range 98-200	**188.33±20.42(188)**<br>**range 130-230**
Global Epilepsy items	25.00±14.96(23)<br>range 0-60	54.05±17.84(57)<br>range 0-86	82.75±19.46(82)<br>range 42-135	**103.90±27.44(100)**<br>range 45-170
**Prenatal/Neonatal**				
**IUGR**	17/21 (80.95%)	38/41 (92.68%)	52/53(98.11%)	**21/21(100%)**
**Medro faillure**	16/21(76.19%)	33/40 (82.50%)	46/53(86.79%)	**19/21(90.48%)**
**Gestational week**	**38.52±1.56 (38)**<br>**range 35-40**	37.06±2.25(37)<br>range 28-41	36.26±2.46(37)<br>range 28-42	35.22±5.03(37)<br>range 17-41
**Weight at birth (gr)**	**2465.00±559.30 (2350)**<br>**range 1190-3170**	2109.39±379.56(2075)<br>range 1400-3200	1863.57±384.55(1840)<br>range 900-3090	1702.48±486.62(1760)<br>range 440-2420
**height at birth (cm)**	**45.14±4.50(45)**<br>**range 33-52**	43.89±2.73(44)<br>range 45-48	43.79±2.99(44)<br>range 35-51	42.09±4.55(42)<br>range 29.5-48
**OFC at birth (cm)**	**32.74±1.94(32.5)**<br>**range 30-36**	31.03±2.75(31)<br>range 21-36	31.67±2.53(31)<br>range 28-39	29.52±3.42(31)<br>range 19.5-32
**EPILEPSY**				
**Seizures**	13/21(61.90%)	39/41 (95.12%)	**53/53(100%)**	**21/21(100.00%)**
**Age of seizures (months)**	**11.57±11.38 (11.5)**<br>**range 0.01-36**	9.93±6.02 (9)<br>range 0-30	9.99±5.30 (9)<br>range 2-30	7.09±4.15 (8)<br>range 0.01-14
**Seizures w fever**	11/20(55.00%)	29/40 (72.50%)	42/53 (79.24%)	13/21 (61.90%)
**Seizures w/o fever**	3/20(15.00%)	22/40 (55.00%)	37/53 (69.80%)	**19/21(90.47%)**
**Status**	3/20(15.00%)	15/40 (37.50%)	**46/53(86.79%)**	16/21 (76.19%)
**Number status**	0.85±2.64 (0)<br>range 0-11	1.18±3.30 (0)<br>range 0-20	**5.13±9.15 (2)**<br>**range 0-55**	3.48±3.08 (3)<br>range 0-10
**Status to ICU**	2/20(10.00%)	8/40 (20.00%)	**35/53(66.03%)**	13/21 (61.90%)
**AEDs**	11/20(55.00%)	33/40 (82.50%)	**51/53 (96.22%)**	**20/21(95.23%)**

**Number of AEDs**	1.22±1.34 (1)<br>range 0-5	1.63±1.21 (1)<br>range 0-4	2.62±1.37 (2)<br>range 0-6	**2.81±1.29 (3)**<br>**range 0-5**
**Monotherapy**	6/20(30.00%)	**26/40 (65.00%)**	24/53 (45.28%)	7/21 (33.33%)
**Max number of AEDs used simultaneously**	0.85±0.81 (1)<br>range 0-2	1.33±0.76 (1)<br>range 0-3	1.81±0.71 (2)<br>range 0-3	**2.29±1.15 (2)**<br>**range 0-5**
**Took drug for epilepsy not now**	**7/20 (35.00%)**	8/40 (20.00%)	4/53 (7.54%)	1/21(4.76%)
**Crisis control (0-6)**	**5.24±0.77 (5)**<br>**range 3-6**	3.95±1.52 (5)<br>range 1-6	3.40±1.45 (3)<br>range 1-5	1.95±1.07 (2)<br>range 0-4
**MOTOR**				
Able to support head	**21/21(100%)**	37/40 (92.50%)	48/53 (90.56%)	15/21 (71.42%)
Able to seat	**21/21(100%)**	32/41 (78.04%)	39/53 (73.58%)	10/21 (47.60%)
Able to seat unaided	**21/21(100%)**	29/41 (70.73%)	34/53 (64.15%)	7/21 (33.33%)
Able to walk with help	**20/21(95.23%)**	26/41 (63.41%)	25/53 (47.16%)	6/21 (28.50%)
Able to walk unaided	**18/21(85.71%)**	17/41 (41.46%)	14/53 (26.41%)	1/21(4.76%)
Able to eat unaided	**15/21(71.42%)**	9/41 (21.95%)	12/53 (22.64%)	1/21(4.76%)
**COGNITIVE**				
**Non-sphincter control**	10/21(47.61%)	32/41 (78.04%)	46/53 (86.79%)	**18/21(85.71%)**
**Able to communicate with environment**	**21/21(100%)**	37/41 (90.24%)	49/53 (92.45%)	16/21 (76.19%)
**Able to communicate with alternative tools**	**20/21(95.23%)**	29/41 (70.73%)	37/53 (69.81%)	8/21 (38.09%)
**Able to say some words**	**17/21(80.95%)**	14/41 (34.14%)	13/53 (24.53%)	2/21 (9.52%)
**Able to make short sentences**	**9/21(42.85%)**	7/41 (17.07%)	3/53 (5.66%)	0/21 (0%)
**COMORBIDITY**				
**C-gastrostomy**	0/21(0%)	2/41 (4.87%)	7/53 (13.20%)	**6/21 (28.57%)**

**Cardiovascular problems**	4/21(19.04%)	14/41 (34.14%)	24/53 (45.28%)	**18/21(85.71%)**
**Nephro-urogenital anomalies**	7/21(33.33%)	18/41 (43.90%)	32/53 (60.38%)	**15/21 (71.42%)**
**Ophthalmological probl.**	7/21(33.33%)	19/41 (46.34%)	36/53 (67.92%)	**15/21 (71.42%)**
**Auditive problems**	6/21(28.57%)	9/41 (21.95%)	**31/53 (58.49%)**	10/21 (47.60%)
**Recurrent ait tract infections**	8/21(38.09%)	22/41 (53.65%)	34/53 (64.15%)	**15/21 (71.42%)**
**Brain anomalies by MRI**	4/19 (21.05%)	21/41 (51.21%)	34/53 (64.15%)	**14/21 (66.66%)**
**Surgeries**	9/21(42.85%)	20/39 (51.28%)	30/53 (56.60%)	**13/21 (61.90%)**
**SOCIAL**				
**a familiy member quit job to care**	8/21 (38.09%)	21/41 (51.21%)	32/53 (60.38%)	**16/21 (76.19%)**
**DEVELOPMENTAL**				
**Motor milestones (0-6)** **(*motor delay*)**	**5.38±1.02 (6)** **range 2-6**	3.61±1.99 (4) range 0-6	3.04±1.90 (3) Range 0-6	1.90±1.51 (2) range 0-4
**Cognitive milestones** **(0-5)** **(*cognitive delay*)**	**3.86±1.15 (4)** **range 2-5**	2.68±144 (3) range 0-5	2.38±0.96 (2) range 0-5	1.76±0.94 (2) range 0-3

Mb, megabases. AU, arbitrary units. DD, developmental delay. Sph/Lat, Spanish/Latin American. IUGR, intrauterine growth restriction. ICU, intensive unit care. OFC, head circumference. AEDs, anti-epileptic drugs. *GFAP, global functional assessment of the patient; MRI, magnetic resonance imaging.*
